# Drug Transporter Expression and Activity in Human Hepatoma HuH-7 Cells

**DOI:** 10.3390/pharmaceutics9010003

**Published:** 2016-12-28

**Authors:** Elodie Jouan, Marc Le Vée, Claire Denizot, Yannick Parmentier, Olivier Fardel

**Affiliations:** 1 Institut de Recherches en Santé, Environnement et Travail (IRSET), UMR INSERM U1085, Faculté de Pharmacie, 2 Avenue du Pr Léon Bernard, 35043 Rennes, France; elodie.jouan@gmail.com (E.J.); marc.levee@free.fr (M.L.V.); 2 Centre de Pharmacocinétique, Technologie Servier, 25–27 Rue Eugène Vignat, 45000 Orléans, France; claire.denizot@servier.com (C.D.); yannick.parmentier@servier.com (Y.P.); 3 Pôle Biologie, Centre Hospitalier Universitaire, 2 rue Henri le Guilloux, 35033 Rennes, France

**Keywords:** hepatoma, hepatocytes, HuH-7, drug transporters, MRP2

## Abstract

Human hepatoma cells may represent a valuable alternative to the use of human hepatocytes for studying hepatic drug transporters, which is now a regulatory issue during drug development. In the present work, we have characterized hepatic drug transporter expression, activity and regulation in human hepatoma HuH-7 cells, in order to determine the potential relevance of these cells for drug transport assays. HuH-7 cells displayed notable multidrug resistance-associated protein (MRP) activity, presumed to reflect expression of various hepatic MRPs, including MRP2. By contrast, they failed to display functional activities of the uptake transporters sodium taurocholate co-transporting polypeptide (NTCP), organic anion-transporting polypeptides (OATPs) and organic cation transporter 1 (OCT1), and of the canalicular transporters P-glycoprotein and breast cancer resistance protein (BCRP). Concomitantly, mRNA expressions of various sinusoidal and canalicular hepatic drug transporters were not detected (NTCP, OATP1B1, organic anion transporter 2 (OAT2), OCT1 and bile salt export pump) or were found to be lower (OATP1B3, OATP2B1, multidrug and toxin extrusion protein 1, BCRP and MRP3) in hepatoma HuH-7 cells than those found in human hepatocytes, whereas other transporters such as OAT7, MRP4 and MRP5 were up-regulated. HuH-7 cells additionally exhibited farnesoid X receptor (FXR)- and nuclear factor erythroid 2-related factor 2 (Nrf2)-related up-regulation of some transporters. Such data indicate that HuH-7 cells, although expressing rather poorly some main hepatic drug transporters, may be useful for investigating interactions of drugs with MRPs, notably MRP2, and for studying FXR- or Nrf2-mediated gene regulation.

## 1. Introduction

Hepatic drug transporters, belonging to the solute carrier (SLC) or ATP-binding cassette (ABC) transporter families, are now recognized as major actors of drug hepatobiliary elimination [1]. They are thus involved in drug uptake from blood at the sinusoidal pole of hepatocytes and in their biliary secretion at the canalicular pole of hepatocytes [2]. In addition, some sinusoidal ABC transporters mediate transport back of drugs or drug metabolites into the blood, for a secondary renal elimination [3]. Modulation of hepatic drug transporter expression or activity, which may occur in response to drug treatment or to various physiological or pathological factors [4,5,6,7,8], is thus susceptible to alter pharmacokinetics of drugs in a major way and to lead to drug-drug interactions [9,10,11]. Analysis of potential interactions of new drugs with some hepatic transporters is consequently now recommended by drug regulatory agencies [12]. 

In vitro hepatic models represent important tools for the study of human hepatic drug transporters, including analysis of potential interactions of drugs with these transporters [13,14]. In this context, human hepatocytes, especially sandwich-cultured hepatocytes, are likely the gold standard [15,16]. However, their availability is scarce and the cost is rather important for human hepatocyte cultures of commercial origin. Human hepatoma cell lines may represent a convenient and cheaper alternative to the use of human hepatocytes [17,18]. As an example, the well-differentiated hepatoma HepaRG cell line has been shown to exhibit notable functional expression of several SLC and ABC transporters, including organic anion-transporting polypetide (OATP/*SLCO*) 1B1 (*SLCO1B1*), organic cation transporter 1 (OCT1/*SLC22A1*), sodium taurocholate co-transporting polypeptide (NTCP/*SLC10A1*), P-glycoprotein (P-gp), encoded by multidrug resistance gene 1 (MDR1)/*ABCB1* gene, and multidrug resistance-associated protein (MRP/*ABCC*) 2 (*ABCC2*) [19,20]. However, HepaRG cells only rather poorly express some important hepatic transporters like OATP1B3 (*SLCO1B3*) or bile salt export pump (BSEP/*ABCB11*) [19]. Moreover, the culture conditions for getting differentiated HepaRG cells, i.e., the addition of dimethylsulfoxide and the culture time (at least four weeks) [21], may be not convenient for some transporter studies. In addition, the HepaRG cell line is patented, which precludes its free use for commercial purpose. Other hepatic cell lines may also be interesting for drug transporter studies. This is notably the case for the hepatoma HuH-7 cell line, already used for analyzing regulation of the ABC transporters MRP2 [22], BSEP [23] and P-gp [24], and of the SLC transporter organic anion transporter (OAT) 7 (*SLC22A9*) [25]. Extensive and accurate characterization of drug transporters in HuH-7 cells is, however, lacking. The present study was, therefore, designed to analyse expression, activity and regulation of main hepatic drug transporters in HuH-7 cells.

## 2. Materials and Methods

### 2.1. Chemicals and Reagents

Rhodamine 123, probenecid, verapamil, fumitremorgin C, rifampicin, phenobarbital, chenodeoxycholic acid (CDCA) and tert-butylhydroquinone (tBHQ) were provided by Sigma-Aldrich (Saint-Quentin Fallavier, France), whereas carboxy-2,7-dichlorofluorescein (CF) diacetate and Hoechst 33342 were from Life Technologies (Saint Aubin, France) and 2,3,7,8-tetrachlorodibenzo-*p*-dioxin (TCDD) from Cambridge Isotope Laboratories (Cambridge, UK). [^3^H(G)] taurocholic acid (sp. act. 5.0 Ci/mmol), [6,7-^3^H(N)] estrone-3-sulfate (E3S) (sp. act. 54.0 Ci/mmol) and [1-^14^C] tetra-ethylammonium (TEA) (sp. act. 3.5 mCi/mmol) were supplied by Perkin-Elmer (Boston, MA, USA). Mouse monoclonal antibodies against P-gp (clone C219), MRP2 (clone M2III-6) and MRP3 (*ABCC3*) (clone M3II-9) were from Alexis Biochemicals (Lausen, Switzerland), whereas rabbit antibody against the p38 mitogen-activated protein kinase (MAPK) was provided by Santa Cruz Biotechnology (Dallas, TX, USA). Mouse monoclonal antibody against MRP4 (*ABCC4*) and rabbit polyclonal antibody against MRP5 (*ABCC5*) were purchased from Abcam (Paris, France). All other chemicals and reagents were commercial products of the highest purity available. 

### 2.2. Cell Culture

The human hepatoma cell line HuH-7, established from a well-differentiated hepatocarcinoma [26], was cultured in Dulbecco’s modified Eagle medium (DMEM, Life Technologies), supplemented with 10% (*v*/*v*) fetal calf serum, 100 IU/mL penicillin and 100 μg/mL streptomycin. HuH-7 cells were routinely used at confluency for transporter studies. Human hepatocytes were obtained from adult donors undergoing hepatic resection for primary and secondary tumors or other pathologies, via the Biological Resource Center (University Hospital, Rennes, France); they were isolated by enzymatic dissociation of histologically-normal liver fragments [27] and primary cultured as previously described [28]. P-gp-overexpressing mammary MCF7R cells [29] were cultured in DMEM, supplemented with 10% (*v*/*v*) fetal calf serum, 100 IU/mL penicillin and 100 μg/mL streptomycin, as previously described [30,31]. Breast cancer resistance protein (BCRP/*ABCG2*)- transfected HEK 293 cells [32], kindly donated by X. Decleves (Faculty of Pharmacy, University Paris-Descartes, Paris, France), were cultured in DMEM supplemented with 10% (*v*/*v*) fetal calf serum, 100 IU/mL amoxicillin, 100 μg/mL erythromycin and 2 mg/mL G418 [30].

### 2.3. RNA Isolation and Analysis

Total RNAs were extracted using the TRI reagent (Sigma-Aldrich). RNA, whose quality was controlled by determining the 260/280 nm absorbance ratio, was then subjected to reverse transcription-quantitative polymerase chain reaction (RT-qPCR), using the RT kit from Applied Biosystems (Foster City, CA, USA), the fluorescent dye SYBR Green methodology and a CFX384 real-time PCR system (Bio-Rad, Hercules, CA, USA), as described previously [33]. Gene-specific primers for drug transporters, aryl hydrocarbon receptor (AhR), nuclear factor erythroid 2-related factor 2 (Nrf2) and 18S rRNA were exactly as already reported [19,34,35,36]; other primers were: OAT7 sense, AGGTTTGGGAGAAGGTTCGT, OAT7 antisense, TTGCCCACTCGGCTATTAAC, pregnane X receptor (PXR) sense, CCAGGACATACACCCCTTTG, PXR antisense, CTACCTGTGATGCCGAACAA, constitutive androstane receptor (CAR) sense, TGATCAGCTGCAAGAGGAGA, CAR antisense, AGGCCTAGCAACTTCGCATA, farnesoid X receptor (FXR) sense, GGAGGATCAAAGGGGATGA, and FXR antisense, CAGTTGCCCCCGTTTTTAC. The specificity of each gene amplification was verified at the end of qPCR reactions through analysis of dissociation curves of the PCR products. Amplification curves were analyzed with CFX Manager software (Bio-Rad), using the comparative cycle threshold method. Relative quantification of the steady-state target mRNA levels was calculated after normalization of the total amount of cDNA tested to the 18S rRNA endogenous reference using the 2^(−ΔΔCt)^ method. Data were finally expressed in arbitrary units relative to 18S rRNA content as described previously [34] or, alternatively, for transporter regulation studies, as percentages of mRNA expression found in control untreated cells.

### 2.4. Western-Blot Analysis

Cellular protein extracts were prepared as already described [37]. Protein lysates were then separated on polyacrylamide gel and electrophoretically transferred onto Protan^®^ nitrocellulose membranes (Whatman GmbH, Dassel, Germany). After blocking in Tris-buffered saline containing 4% bovine serum albumin, membranes were incubated overnight at 4 °C with primary antibodies directed against target proteins. Peroxidase-conjugated monoclonal antibodies were thereafter used as secondary antibodies. After washing, immunolabeled proteins were finally visualized by chemiluminescence. Gel loading and transfer were checked by immunostaining membranes with antibody against the p38 MAPK.

### 2.5. Drug Transport Assay

Activities of the sinusoidal SLC uptake transporters NTCP, OATPs and OCT1 were measured through determining intracellular accumulation of reference substrates for 10 min, using a well-defined transport medium, as previously reported [38]. Briefly, for NTCP activity, cells were incubated at 37 °C with 40.0 nM [^3^H] taurocholate, in the absence or presence of sodium; for OATP activity, cells were incubated at 37 °C for 10 min with 3.8 nM [^3^H] E3S, in the absence or presence of 2 mM probenecid, used as a reference OATP inhibitor [39]; for OCT1 activity, cells were incubated at 37 °C with 28.6 μM [^14^C] TEA for 10 min, in the absence or presence of 50 µM verapamil, used as a reference OCT1 inhibitor [40]. After washing in phosphate-buffered saline (PBS), cells were lysed and intracellular accumulation of substrates was determined through scintillation counting. Data were expressed as amount of intracellular substrate/mg protein.

Activities of the ABC canalicular transporters P-gp, MRP2 and BCRP were determined by measuring intracellular accumulation or retention of reference fluorescent substrates, as previously described [38]. Briefly, for P-gp activity, cells were incubated with 5.25 µM rhodamine 123 for 30 min at 37 °C in the presence or absence of 50 µM verapamil, used here as a reference P-gp inhibitor [41]; for MRP activity, cells were first loaded with 3 µM CF diacetate for 30 min at 37 °C, then washed with PBS and reincubated in CF diacetate-free medium for 60 min at 37 °C in the absence or presence of 2 mM probenecid, used here as a reference MRP inhibitor [42]; for BCRP activity, cells were first loaded with 16.2 µM Hoechst 33342 for 30 min at 37 °C, then washed with PBS and reincubated in Hoechst 33342-free medium for 90 min at 37 °C in the absence or presence of 10 µM fumitremorgin C, used here as a reference BCRP inhibitor [43]. Cells were finally lysed in distilled water and intracellular levels of fluorescence dyes were quantified by spectrofluorimetry using a SpectraMax Gemini SX spectrofluorimeter (Molecular Devices, Sunnyvale, CA, USA) (excitation and emission wavelengths were 485 and 535 nm, respectively, for rhodamine 123 and CF, and 355 and 460 nm, respectively, for Hoechst 33342). Data were expressed as fluorescence arbitrary units/mg protein. 

### 2.6. Data Analysis

Experimental data were routinely expressed as means ± SEM from at least three independent experiments. Statistical analysis of quantitative data was performed by Student’s *t*-test, which is applicable to small size samples [44], or analysis of variance (ANOVA) followed by Dunnett’s or Tukey’s post-hoc test, using the GraphPad Prism software (GraphPad software, La Jolla, CA, USA). The criterion of significance was *p* < 0.05. 

## 3. Results

### 3.1. Expression of Drug Transporter mRNAs in HuH-7 Cells

**Figure 1:**
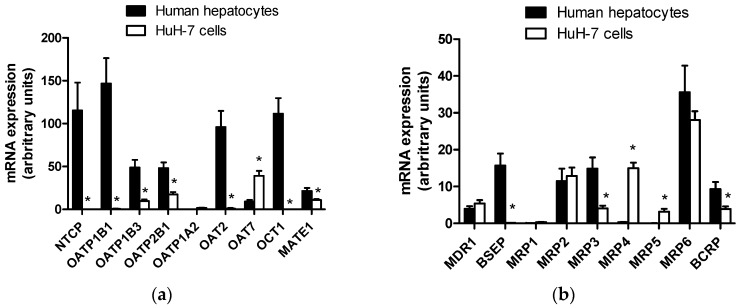
Solute carrier SLC (**a**) and ATP-binding cassette (ABC) (**b**) drug transporter mRNA expression in HuH-7 cells. Drug transporter mRNA expression was determined by RT-qPCR in human hepatoma HuH-7 cells and in freshly isolated human hepatocytes. Data are expressed as arbitrary units and are the means + SEM from at least three independent assays (HuH-7 cells) or from three independent populations (human hepatocytes). * *p* < 0.05 when compared to transporter mRNA expression level found in freshly isolated human hepatocytes (Student’s *t*-test).

Expression levels of hepatic drug transporter mRNAs were determined in HuH-7 cells and compared to those found in freshly isolated human hepatocytes. As shown in Figure 1a, SLC transporters were very poorly expressed in HuH-7 cells comparatively to hepatocytes. Indeed, HuH-7 cells failed to express the sinusoidal uptake transporters NTCP, OATP1B1 (*SLCO1B1*), OAT2 (*SLC22A7*) and OCT1, which, by contrast, were highly expressed in human hepatocytes, in agreement with previous data [45]. OATP1A2 (*SLCO1A2*) mRNAs were also not detected in HuH-7 cells, knowing, however, that they were similarly not present in human hepatocytes (Figure 1a). With respect to the sinusoidal transporters OATP1B3 and OATP2B1 (*SLCO2B1*) and to the canalicular SLC transporter multidrug and toxin extrusion protein 1 (MATE) 1 (*SLC47A1*), they were expressed in HuH-7 cells, but at significantly lower levels than those found in hepatocytes. Finally, OAT7 was the only SLC transporter more expressed in HuH-7 cells than in human hepatocytes (Figure 1a).

Some ABC transporters, such as MDR1, MRP2 and MRP6 (*ABCC6*), exhibited similar levels of mRNA expression in HuH-7 cells and hepatocytes (Figure 1b), whereas MRP1 (*ABCC1*) was not present, or only at a very low level, in the two types of hepatic cells. BSEP was similarly not present in HuH-7 cells, but was highly expressed in human hepatocytes (Figure 1b). Levels of MRP3 and BCRP mRNAs were also significantly higher in hepatocytes than in HuH-7 cells. By contrast, MRP4 and MRP5, not detected or only at very low level in human hepatocytes, were obviously upregulated in HuH-7 cells at mRNA level (Figure 1b). 

### 3.2. Drug Transporter Protein Expression in HuH-7 Cells

**Figure 2:**
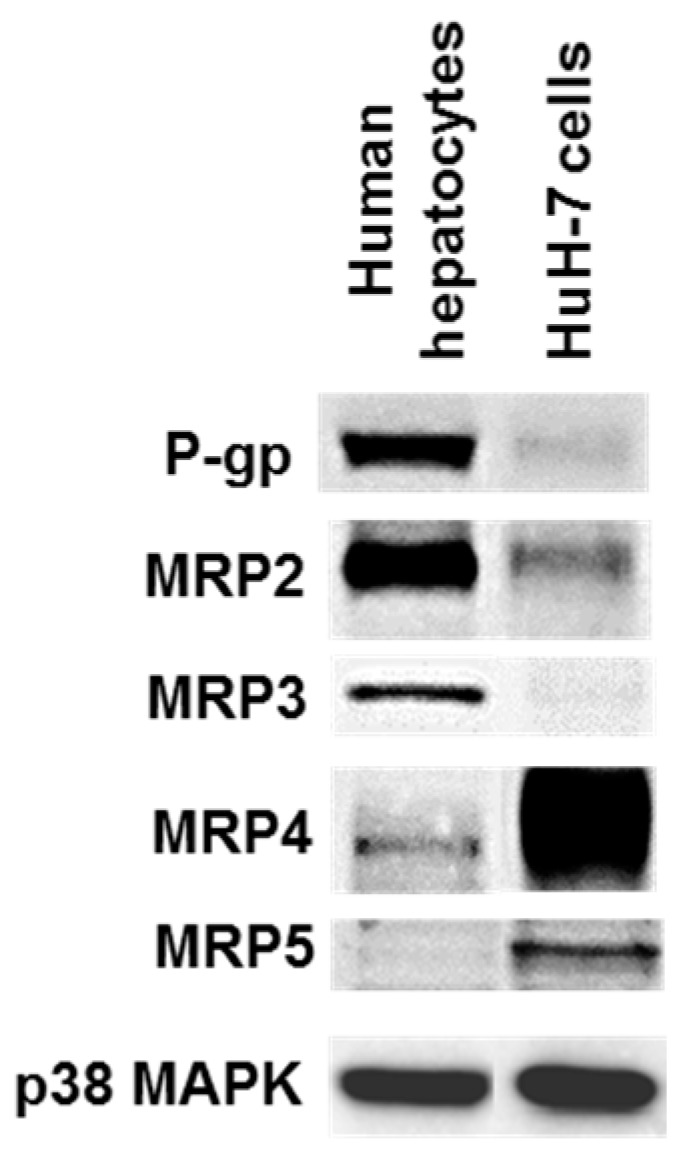
Drug transporter protein expression in HuH-7 cells. Protein content was determined in HuH-7 cells and freshly isolated human hepatocytes by Western-blotting. Data shown are representative of three independent assays (HuH-7 cells) or of three independent populations (human hepatocytes). P-gp, P-glycoprotein; MRP, multidrug resistance-associated protein; MAPK, mitogen-activated protein kinase.

HuH-7 cells were found to express P-gp, MRP2, MRP4 and MRP5 at detectable protein level (Figure 2). Expression of P-gp and MRP2 were, however, weaker than those found in human hepatocytes, wheras that of MRP4 and MRP5 was much higher. HuH-7 cells additionnally failed to obviously express MRP3, that, by contrast, was clearly detected in human hepatocytes (Figure 2). 

### 3.3. Drug Transporter Activities in HuH-7 Cells

**Figure 3:**
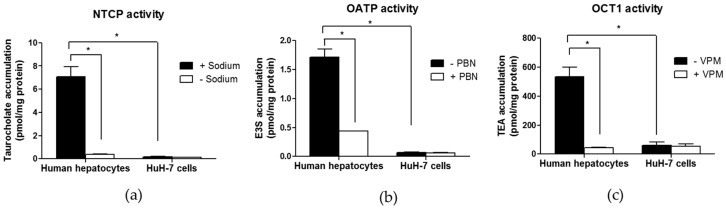
Activities of the sinusoidal SLC uptake transporters sodium taurocholate co-transporting polypeptide (NTCP) (**a**), organic anion-transporting polypetides (OATPs) (**b**) and organic cation transporter 1 (OCT1) (**c**) in HuH-7 cells. HuH-7 cells and primary human hepatocytes were incubated with taurocholate in the presence or absence of sodium (NTCP activity), with E3S in the absence or presence of 2 mM probenecid (PBN) (OATP activity) or with tetra-ethylammonium (TEA) in the absence or presence of 50 µM verapamil (VPM) (OCT1 activity). Intracellular accumulations of substrates were next determined by scintillation counting. Data are the means + SEM from three independent assays (HuH-7 cells) or from three independent populations (human hepatocytes). * *p* < 0.05 (ANOVA followed by Tukey’s post-hoc test).

With respect to activity of sinusoidal SLC uptake transporters, HuH-7 cells failed to exhibit uptake of the NTCP substrate taurocholate, of the OATP substrate E3S or of the OCT1 substrate TEA (Figure 3). By contrast, primary human hepatocytes exhibited sodium-dependent uptake of taurocholate, probenecid-sensitive uptake of E3S and verapamil-sensitive uptake of TEA (Figure 3). NTCP, OATP and OCT1 activities have, therefore, been detected in primary human hepatocytes, in agreement with previous data [28,38], but not in HuH-7 cells.

**Figure 4:**
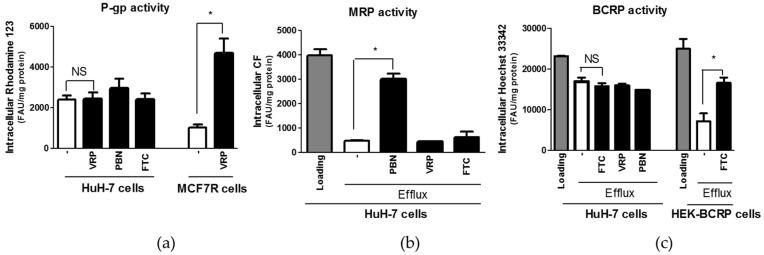
Activities of the canalicular ABC efflux transporters P-glycoprotein (P-gp) (**a**), multidrug resistance-associated proteins (MRPs) (**b**) and breast cancer resistance protein (BCRP) (**c**) in HuH-7 cells. For P-gp activity, HuH-7 were incubated for 30 min with the P-gp substrate rhodamine 123 in the absence or presence of the ABC transporter modulators verapamil (VPM) (50 µM), probenecid (PBN) (2 mM) or fumitremorgin C (FTC) (10 µM); MCF7R cells were used in parallel as control P-gp-positive cells. For MRP activity, HuH-7 cells, initially loaded with the MRP substrate carboxy-2,7-dichlorofluorescein (CF) used under its diacetate ester form for 30 min, were reincubated in CF diacetate-free medium cells for 60 min in the absence or presence of ABC transporter modulators. For BCRP activity, HuH-7 cells, loaded with the BCRP substrate Hoechst 33342 for 30 min, were reincubated in Hoechst 33342-free medium for 90 min in the presence or absence of ABC transporter modulators; BCRP-transfected HEK 293 cells were used in parallel as control BCRP-positive cells. Intracellular levels of fluorescent substrates were next determined by spectrofluorimetry. Data, expressed as fluorescence arbitrary unit (FAU)/mg protein, are the means + SEM of three independent assays. * *p* < 0.05 and NS, not statistically significant (ANOVA followed by Tukey’s post-hoc test or Student’s *t*-test).

With respect to activity of P-gp, HuH-7 cells failed to exhibit verapamil-sensitive accumulation of rhodamine 123, in contrast to MCF7R cells, used here as P-gp-positive control cells (Figure 4). These data indicate that P-gp activity can not be detected in HuH-7 cells. In the same way, the hepatoma cells failed to display fumitremorgin C-sensitive retention of Hoechst 33342, in contrast to BCRP-transfected HEK293 cells (Figure 4). These results do not argue in favor of a detectable BCRP activity in HuH-7 cells. By contrast, retention of the MRP substrate CF in HuH-7 cells was markedly increased by the MRP inhibitor probenecid, but not by verapamil or fumitremorgin C (Figure 4), which is fully convenient with the fact that HuH-7 cells exhibit MRP efflux activity.

### 3.4. Drug Transporter mRNA Regulation in HuH-7 Cells

**Figure 5:**
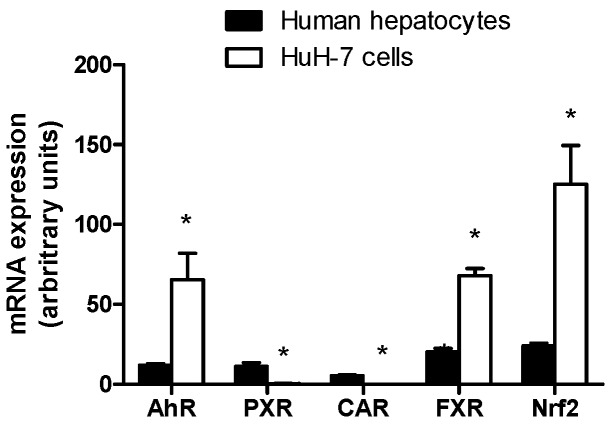
Nuclear receptor mRNA expression in HuH-7 cells. mRNA expression of the receptors aryl hydrocarbon receptor (AhR), pregnane X receptor (PXR), constitutive androstane receptor (CAR), farnesoid X receptor (FXR) and nuclear factor erythroid 2-related factor 2 (Nrf2) was determined by RT-qPCR in human hepatoma HuH-7 cells and in freshly isolated human hepatocytes. Data are expressed as arbitrary units and are the means + SEM from at least three independent assays (HuH-7 cells) or from three independent populations (human hepatocytes). * *p* < 0.05 when compared to nuclear receptor mRNA expression level found in freshly isolated human hepatocytes (Student’s *t*-test).

Expression levels of nuclear receptors such as AhR, PXR, CAR, FXR and Nrf2, that mediate major regulations of drug transporter expression [46,47,48,49,50,51], were analyzed in HuH-7 cells by RT-qPCR and compared to those found in freshly isolated human hepatocytes. As shown in Figure 5, HuH-7 cells displayed enhanced mRNA expression of AhR, FXR and Nrf2 comparatively to hepatocytes. By contrast, the hepatoma cells failed to exhibit mRNA expression of PXR and CAR (Figure 5).

**Figure 6:**
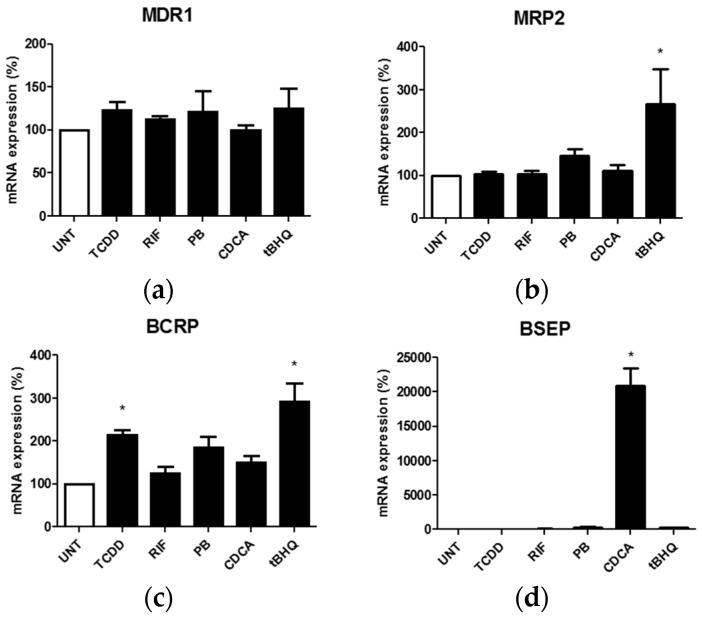
Effects of prototypical activators of nuclear receptors on multidrug resistance gene 1 (MDR1) (**a**); multidrug resistance-associated protein 2 (MRP2) (**b**); breast cancer resistance protein (BCRP) (**c**); and bile salt export pump (BSEP) (**d**) mRNA expression in HuH-7 cells. HuH-7 cells were either untreated (UNT) or exposed for 24 h to 10 nM 2,3,7,8-tetrachlorodibenzo-*p*-dioxin (TCDD), 25 µM rifampicin (RIF), 2 mM phenobarbital (PB), 100 µM chenodeoxycholic acid (CDCA) or 80 µM tert-butylhydroquinone (tBHQ). MDR1, MRP2, BCRP and BSEP mRNA expressions were then analyzed by RT-qPCR. Data are expressed as % of transporter expression found in untreated control cells, arbitrarily set at 100%; they are the means + SEM of three independent experiments. * *p* < 0.05 when compared to control untreated cells (ANOVA followed by Dunnett’s post-hoc test).

Regulation of reference transporters in response to prototypical activators of nuclear receptors was finally investigated in HuH-7 cells. TCDD, a reference activator of AhR, was found to induce BCRP mRNA expression, whereas CDCA, an activator of FXR, markedly increased BSEP mRNA levels (Figure 6), in agreement with known regulations of these transporters by AhR- and FXR-related pathways [46,50]. The Nrf2 activator tBHQ enhanced mRNA expression of both MRP2 and BCRP (Figure 6). By contrast, rifampicin, a reference activator of PXR, and phenobarbital, an activator of the CAR signaling pathway, failed to upregulate MRP2 mRNA expression in HuH-7 cells (Figure 6), knowing that MRP2 has been previously shown to be induced by these chemicals in primary hepatocytes in a PXR- or CAR-dependent manner [47]. In the same way, mRNA expression of MDR1, a well-established target of the PXR pathway [49,52], was not impaired by rifampicin in HuH-7 cells (Figure 6).

## 4. Discussion

In the present study, we have analyzed functional expression of various hepatic drug transporters in hepatoma HuH-7 cells. Among drug transport activities, efflux of the MRP substrate CF was easily detected in HuH-7 cells. This secretion of this fluorescent dye was inhibited by probenecid, a reference MRP inhibitor not known to interact with P-gp or BCRP, but not by the P-gp inhibitor verapamil or by the BCRP inhibitor fumitremorgin C, thus demonstrating that it was related to MRP activity, and not to P-gp or BCRP activities. Potential implication of the efflux pump BSEP can additionally be discarded because BSEP mRNA expression was not detected in HuH-7 cells and also because CF has not been reported to be transported by BSEP. By contrast, CF has been previously shown to be handled by several MRPs such as MRP1, MRP2, rat Mrp3 and MRP5 [53,54,55], which agrees with the fact that many substrates, and also many inhibitors, are shared by MRP isoforms. Because HuH-7 cells did not exhibit MRP1 mRNA expression, like human hepatocytes [56], and also displayed no obvious expression of MRP3 protein associated to reduced MRP3 mRNA levels when compared to hepatocytes, an involvement of MRP1 or MRP3 in CF efflux from HuH-7 cells is unlikely. A role for MRP4, markedly up-regulated in HuH-7 cells at mRNA and protein level, may also putatively be discarded, according to the lack of transport of the dye by this transporter [54]. A contribution of MRP6, a transporter primarily involved in Pseudoxanthoma elasticum, a genetic disease affecting elastic fibers in the body, and whose mRNA expression is well-preserved in HuH-7 cells when compared to human hepatocytes, is also unlikely because there is little evidence that this transporter handles efficiently anionic xenobiotics [57]. By contrast, a major implication of MRP2 has to be considered, owing to the fact that this transporter is expressed at mRNA and protein level in HuH-7 cells, even if MRP2 protein expression is reduced when compared to that found in human hepatocytes, which may reflect differential translational/post-translational regulation of MRP2 in HuH-7 cells versus human hepatocytes. Moreover, CF has been previously shown to be a reference relevant probe to investigate MRP2 activity in hepatocytes [58]. Additionnally, MRP5, up-regulated at mRNA and protein level in HuH-7 cells, may contribute to CF efflux. Taken together, these data suggest that HuH-7 cells are convenient for studying MRP activity, notably for investigating inhibitors or substrates of MRP2. These cells may also be useful for analyzing MRP4 activity, owing to their high levels of mRNA and protein expression of this ABC transporter. Such an up-regulation of MRP4 has been described in other hepatoma cells such as HepG2 and HepaRG cells [19,59] and additionally occurs in end-stage liver diseases [60], suggesting that it may be linked to alteration of the hepatic differentiated status. The fact that primary cultured rodent hepatocytes, that exhibit loss of differentiated functions with time in culture [61], concomitantly display up-regulation of Mrp4 expression [62] fully supports this hypothesis.

In contrast to MRP activity, those related to the uptake transporters NTCP, OATPs and OCT1 were not detected in HuH-7 cells. Such data are consistent with the fact that HuH-7 cells displayed no, or only reduced, mRNA expressions of NTCP, OATP1B1, OATP1B3, OATP2B1, OATP1A2 and OCT1, knowing moreover that protein expression of these transporters remain to be further explored. Similarly, OAT2 mRNA levels were not detected in HuH-7 cells, whereas by contrast, those of OAT7 were up-regulated. This OAT7 mRNA expression likely confirms that HuH-7 cells may represent an adequate model for investigating OAT7 regulation at transcriptional level [25], even if these cells failed to display OAT7 activity, i.e., they did not exhibit uptake of E3S, that is a substrate shared by OATPs and OAT7 [63]. Importantly, the absence of OATP activity in HuH-7 cells may preclude the entry, and by consequence the MRP-mediated efflux, of anionic drugs. The use of HuH-7 cells for analyzing MRP activity may, therefore, theoretically concern only drugs exhibiting some degree of hydrophobicity, enabling initial passive diffusion across the plasma membrane for entering HuH-7 cells. Such a criteria may fortunately be fullfilled by most of drugs, for which passive and carrier-mediated transport across the plasma membrane coexist [64].

HuH-7 cells also failed to display detectable activity of P-gp and BCRP, which agrees with the fact that expression of P-gp protein as well as that of BCRP mRNAs were reduced in these hepatoma cells when compared to human hepatocytes. MDR1 mRNA levels were, however, similar in HuH-7 cells and hepatocytes, thus suggesting divergent regulation of P-gp at protein and mRNA levels, possibly due to different P-gp post-transcriptional regulation processes. Other canalicular transporters such as MATE1 and BSEP exhibited reduced mRNA expressions in HuH-7 cells when compared to human hepatocytes, thus fully supporting the conclusion that expression of various main drug transporters is reduced or not detected in hepatoma HuH-7 cells. Moreover, HuH-7 cells failed to display expression of the xenobiotic-sensing receptors PXR and CAR, involved in transporter regulation; they were concomitantly not responsive to prototypical activators of these receptors such as rifampicin and phenobarbital. Taken together, these data suggest that HuH-7 cells are unlikely to represent a convenient alternative to the use of human hepatocytes for studying activity or regulation of most of main hepatic drug transporters. Similarly, the HepG2 cell line, another hepatoma cell line widely used as a surrogate of human hepatocytes in biological and biochemical studies [65], failed to to exhibit expression of major hepatic drug transporters [66] and can, therefore, also not be retained for transport studies. By contrast, the well-differentiated HepaRG cell line displays substantial mRNA expression of hepatic drug transporters, such as NTCP, OATP1B1, OCT1, P-gp and MRP2, and retains signaling ways of transporter regulation [19,20,67]. This cell line remains consequently yet as the hepatoma cell line whose transporter expression profile may be considered as the closest to that found in human hepatocytes, thus likely underlying the growing interest for using it in pharmacological and toxicological studies [68,69]. HepaRG cells, however, exhibit some limits, notably the requirement of dimethylsulfoxide for getting a full differentiated status [21] and their protein expression of most of drug transporters weaker than that found in primary human hepatocytes [70]. These human hepatoma cells can, therefore, likely not correspond to fully relevant surrogates to human hepatocytes [71]. Another promising alternative to primary human hepatocytes and to their scarce availability may be immortalized engineered hepatocyte cell lines, which have been described to retain key differentiated functions of normal hepatocytes, at least for some of them [72]. 

In contrast to signaling pathways linked to PXR and CAR, that related to AhR was active in HuH-7 cells, as demonstrated by mRNA expression of AhR and up-regulation of the drug transporter BCRP in response to TCDD, a prototypical ligand for AhR. This conclusion is fully supported by the fact that TCDD has previously been shown to induce expression of the reference AhR target gene cytochrome P-450 1A1 in HuH-7 cells [73]. FXR-signalling pathway is similarly active in HuH-7 cells, as notably demonstrated by the marked induction of the FXR target BSEP in response to CDCA, a reference ligand for FXR. HuH-7 cells may, therefore, be useful for screening agonistic properties of drugs towards FXR and for studying FXR-related gene regulation, notably FXR-mediated BSEP regulation, as already reported [23,74]. Finally, Nrf2 pathway, notably involved in gene regulation in response to xenobiotic-induced oxidative stress [75], was also preserved in HuH-7 cells, as shown by the induction of the Nrf2 targets MRP2 and BCRP by tBHQ and in agreement with previous data [76]. In this context, however, it is noteworthy that transporter regulations in HuH-7 cells exposed to prototypical activators of nuclear receptors were only investigated at the mRNA level in the present study and remain, therefore, to be validated at the protein level through additional work. 

## 5. Conclusions

The human hepatoma HuH-7 cells were found to exhibit notable MRP activity, likely reflecting, at least partly, MRP2 expression. They additionally retained some transporter regulatory ways, like FXR-related BSEP up-regulation and Nrf2-mediated MRP2 and BCRP induction. They failed, however, to display functional expression of main hepatic drug transporters, like OATPs, OCT1, P-gp and BCRP. Such data, therefore, indicate that HuH-7 cells are likely not relevant as full surrogates of human hepatocytes in drug transport studies, but have to be positively considered for investigating interactions of drugs with MRPs, notably MRP2, and for studying FXR- or Nrf2-related gene regulation.
